# Trait mindfulness and personality characteristics in a microdosing ADHD sample: a naturalistic prospective survey study

**DOI:** 10.3389/fpsyt.2023.1233585

**Published:** 2023-10-16

**Authors:** Eline C. H. M. Haijen, Petra P. M. Hurks, Kim P. C. Kuypers

**Affiliations:** Department of Neuropsychology and Psychopharmacology, Faculty of Psychology and Neuroscience, Maastricht University, Maastricht, Netherlands

**Keywords:** ADHD, microdosing, psychedelics, mindfulness, personality

## Abstract

**Background:**

Microdosing (MD), repeatedly taking psychedelics in small, non-hallucinogenic amounts, has been practiced by individuals to relieve attention deficit hyperactivity disorder (ADHD) symptoms. Generally, adults diagnosed with ADHD have lower levels of mindfulness and differ in personality structure from non-ADHD adults. How MD affects mindfulness and personality in adults with ADHD remains unexplored.

**Aim:**

This study aimed to investigate the effects of 4 weeks of MD on mindfulness and personality traits in adults diagnosed with ADHD and those experiencing severe ADHD symptoms. It was expected that mindfulness and the personality traits conscientiousness, extraversion, agreeableness, and openness would increase and neuroticism would decrease after 4 weeks of MD compared to baseline. It was explored if using conventional ADHD medication alongside MD and/or having comorbidities influenced MD-induced effects.

**Methods:**

An online prospective naturalistic design was used to measure participants before MD initiation and 2 and 4 weeks later. Validated self-report measures were used assessing mindfulness (15-item Five Facet Mindfulness Questionnaire) and personality traits (10-item version of the Big Five Inventory) at three time points.

**Results:**

The sample included *n* = 233, *n* = 66, and *n* = 44 participants at the three time points, respectively. Trait mindfulness, specifically description and non-judging of inner experience, was increased, and neuroticism was decreased after 4 weeks of MD compared to baseline. The remaining personality traits remained unchanged. Using conventional medication and/or having comorbid diagnoses did not change the MD-induced effects on mindfulness and personality traits after 4 weeks.

**Conclusion:**

MD induced changes in otherwise stable traits. Future placebo-controlled studies are warranted to confirm whether these changes occur in a controlled setting.

## 1. Introduction

Attention deficit hyperactivity disorder (ADHD) is one of the most common developmental disorders worldwide; with a prevalence rate of 2.5% in adults (1–3). ADHD is characterized by symptoms of inattention, hyperactivity, and/or impulsivity, with diverse combinations of symptoms possible. Survey research has shown that some adults diagnosed with ADHD report using low, sub-hallucinogenic doses of psychedelic substances repeatedly, referred to as microdosing (MD), to self-treat their symptoms and as such to improve daily life functioning (4–7). Next to ADHD symptoms, which is mainly the focus of intervention studies, it has also been shown that this population has a different personality and trait mindfulness profile than the neurotypical population. Previous research has established a strong link between certain personality traits and mindfulness (8–10) and it has been suggested that MD could potentially alter personality traits and mindfulness in healthy and general population samples [e.g., (6, 11–13)]. It is not yet clear whether MD affects these stable traits in adults with ADHD.

Trait mindfulness can be described as the inherent general tendency to be mindful in daily life, to be able to allocate and maintain attentional resources to the present experience (e.g., being able to pay attention to the sensation of water when taking a shower), and to be non-judgmental and non-reactive toward arising thoughts (e.g., being able to notice distressing thoughts without reacting to them) (14, 15). Mindfulness can broadly be divided into two dimensions: self-regulation of attention and acceptance (16). First, self-regulation of attention is characterized by attending, observing, and becoming aware of one’s thoughts, feelings, and sensations without getting caught in ruminative thought streams. Mindfulness facets that belong to this domain include observation, description, and acting with awareness (14). Second, the acceptance domain involves taking an open, and accepting stance toward one’s observed experience, thereby inhibiting emotional impulsive responses to whatever is observed. Mindfulness facets that belong to this domain include non-judging of inner experience and non-reactivity to inner experience (14). Generally, individuals diagnosed with ADHD scored on average lower on trait mindfulness compared to individuals without an ADHD diagnosis (17). Interestingly, enhanced levels of mindfulness have been reported after MD (6) and mindfulness scores were higher in current and former microdosers compared to MD-naïve controls (11). Though one prospective (survey) study investigating adults (excluding individuals with mood, anxiety, substance use, psychotic or dissociative disorders) did not find a change in mindfulness scores after MD (13), and another prospective study investigating a general population sample could not attribute changes to MD solely (18). However, the effects of MD on mindfulness in individuals diagnosed with ADHD, or individuals experiencing severe ADHD symptoms, have not been investigated yet.

Mindfulness was strongly and positively related to conscientiousness (i.e., being well-organized, responsible, and efficient) and negatively related to neuroticism (i.e., negative affectivity and emotionally unstable) (8–10). While the relationships between mindfulness and conscientiousness and neuroticism have been a consistent finding, the relationships between mindfulness and the other personality traits agreeableness (i.e., compromising with, and trusting others), extraversion (i.e., positive emotionality and socially engaged), and openness (i.e., curious and willing to explore new experiences) (19) were overall positive, yet less strong (8, 9). Individuals diagnosed with ADHD score generally lower on conscientiousness and higher on neuroticism compared to controls (20–24). Though the associations between ADHD and extraversion and agreeableness are less strong, both traits tend to be lower in ADHD (20, 21, 23–26). Openness is generally unrelated to ADHD (20, 23, 24). Interestingly, openmindesness (i.e., openness) was higher and negative emotionality (i.e., neuroticism) was lower in current and former microdosers compared to MD-naïve controls (27). Also, a qualitative interview study reported that participants experienced increases in openness and extraversion following MD, although no explicit mention of other personality traits was made (28). Prospective MD studies have also reported alterations in personality traits after MD. Namely, conscientiousness was increased in healthy adults after MD (29), but other studies did not find this effect in a general population sample (12) and a general sample without individuals diagnosed with a mood, anxiety, substance use, psychotic or dissociative disorder (13). Agreeableness was increased after MD in a general population sample (12), though other studies investigating healthy individuals did not find this effect (13, 29). Neuroticism decreased after MD in healthy adults (29) and a general population sample (12), but was increased in a sample consisting of adults without mood, anxiety, substance use, and psychotic or dissociative disorders (13). If and in which direction personality traits are altered by MD in individuals diagnosed with ADHD and/or experiencing severe ADHD symptoms remains unexplored.

Therefore, the current study aimed to investigate mindfulness and personality traits in individuals diagnosed with ADHD and/or experiencing severe ADHD symptoms before and after self-initiated MD. First, it was expected that trait mindfulness would increase after MD compared to baseline. No hypotheses were formulated regarding the effect of MD on the different mindfulness facets, because of a lack of research investigating this. Second, based on previous studies, conscientiousness, agreeableness, extraversion, and openness were expected to increase after MD. Previous results of MD effects on neuroticism were somewhat conflicting. However, given that neuroticism is generally higher in ADHD and survey studies reporting potential therapeutic effects of MD in ADHD, we expected neuroticism to decrease in the current study. Lastly, it was investigated if using conventional medication alongside MD or having comorbidities alongside ADHD would influence the change in mindfulness and personality traits induced by MD.

## 2. Materials and methods

### 2.1. Study design and participants

The current study was part of a larger study (7), which used a prospective naturalistic design. The current study set out to assess trait mindfulness and personality traits in an ADHD sample at baseline, before MD initiation, and at 2 and 4 weeks later. Adults diagnosed with ADHD and adults without an ADHD diagnosis who experienced ADHD symptoms to the extent that these interfered with daily life were invited to participate in the study. ADHD symptom severity was determined at baseline using the Conners’ Adult ADHD Rating Scale (CAARS-S:SV) (30). Those without an ADHD diagnosis, who had T-scores lower than 65 on all CAARS-S:SV subscales were excluded from all analyses, as T-scores of 65 and above are indicative of clinically elevated symptoms (see section 2.3.4.). Lastly, all participants had the intention to start MD with psychedelics on their initiative to relieve these symptoms and provided informed consent prior to starting the study.

### 2.2. Study procedure

Participants were recruited through an online advertisement that was placed on a website providing information about MD with psychedelics.^1^ Interested individuals could click the link below the advertisement and were subsequently provided with information regarding the study rationale, procedure, and contact details of the researchers to ask questions about the study. The information included the request to sign up for the study between 1 and 3 days before MD initiation to receive the surveys at the correct moments. After providing informed consent, participants were redirected to the baseline measure. When finishing the baseline survey, participants were enrolled in an emailing system, sending links to the following surveys exactly 2 and 4 weeks after completing the baseline survey. All three surveys took between 15 and 20 min to complete. Furthermore, through daily short surveys, participants were asked if they had taken a microdose that day and if yes, what substance and dose they took. These daily surveys were sent the day after completing the baseline survey until the 4-week time point. Data collection started in November 2020 and ended in July 2021. The Ethics Review Committee of Psychology and Neuroscience at Maastricht University approved the study (reference number: ERCPN-215_05_11_2019_A1).

### 2.3. Measures

#### 2.3.1. Demographic information and history of substance use

Demographic information, such as biological sex, gender, age, continent of residence, educational level, and daily occupation, was collected at baseline. Additionally, information about participants’ previous experience with psychedelics (i.e., ayahuasca, DMT, 5-MeO-DMT, LSD, novel lysergamides (e.g., 1P-LSD, ALD-52), psilocybin, Salvia divinorum, ibogaine, and mescaline) in both full and microdoses was collected at baseline.

#### 2.3.2. Experience with mindfulness/meditation

At baseline, participants were asked if they had any experience in the practice of meditation/mindfulness. If this question was answered with ‘yes’, it was asked what meditation/mindfulness tools were used, where they could choose multiple answers from the following options: ‘I followed an online course’, ‘I use(d) a mobile application’, ‘I watch(ed) Youtube videos’, ‘I follow(ed) sessions at a retreat’, or the option to provide an answer through free text entry. Subsequently, it was asked when the last time was that the participant practiced meditation/mindfulness (i.e., ‘more than one year ago’; ‘less than one year ago, more than one month ago’; ‘less than one month ago, more than one week ago’; or ‘within the past seven days’). A variable ‘recent mindfulness’ was created to group individuals based on the recentness of their mindfulness practice (0 = no experience with meditation/mindfulness or practiced it more than 7 days ago; 1 = practiced meditation/mindfulness within the past 7 days), to differentiate respondents who recently practiced meditation/mindfulness from respondents who did not.

#### 2.3.3. Psychiatric and physical diagnoses

Participants were asked if they had been diagnosed by a medical doctor or a therapist with a psychiatric, neurological, or physical disorder, and if so, what these diagnoses were. Pre-set answer options included ‘ADHD’, ‘depression’, ‘anxiety disorder’, ‘substance use disorder’, ‘dyslexia’, ‘autism/Asperger syndrome’, ‘obsessive-compulsive disorder’, ‘bipolar disorder, chronic pain’, ‘cluster headaches’, ‘epilepsy’, ‘migraines’, ‘post-traumatic stress disorder (PTSD)’, ‘schizophrenia’, ‘I do not want to mention’, or the option to provide another answer in a textbox. A variable ‘comorbidity’ was constructed differentiating participants with at least one comorbid diagnosis alongside ADHD from participants without comorbid diagnoses alongside ADHD or without an ADHD diagnosis (0 = only ADHD or no ADHD; 1 = ADHD and at least one other diagnosis).

Respondents who indicated having an ADHD diagnosis were asked at what age they received the diagnosis and if they were currently using prescribed ADHD medication, stopped using it, or never used it. If they indicated to be using prescribed medication, it was asked what type of medication this was (i.e., ‘Adderall (amphetamine)’, ‘Concerta (methylphenidate)’, ‘Dexedrine (amphetamine)’, ‘Focalin (dexmethylphenidate)’, ‘Ritalin (methylphenidate)’, ‘Strattera (atomoxetine hydrochloride)’, ‘I do not want to mention’, or a free text entry). In the case prescribed ADHD medication was discontinued in the past, it was asked what the reasons were for this: ‘it did not relieve my symptoms’, ‘because of psychological side effects’, ‘because of physical side effects’, ‘I do not want to mention’, or the option to provide another reason through free text entry. A variable ‘medication use’ was constructed differentiating participants using conventional medication alongside MD during the study from participants without conventional medication, who were only MD during the study (0 = only MD; 1 = MD and using conventional ADHD medication).

#### 2.3.4. ADHD symptoms

The self-report, short screening version of the Conners’ Adult ADHD Rating Scale (CAARS-S:SV) (30) was used to assess ADHD symptoms at baseline. This 30-item questionnaire assesses the core ADHD symptoms (i.e., inattention and hyperactivity/impulsivity) as well as related problem areas like problems with self-concept. Participants indicated to what extent the items described them on a four-point Likert scale from 0 (not at all, never) to 4 (very much, very frequently). Nine items belong to the *inattention* subscale, capturing problems experienced with attention and containing items such as ‘I lose things necessary for tasks or activities (e.g., to-do lists, pencils, books, or tools)’. Nine items belong to the *hyperactivity/impulsivity* subscale, capturing symptoms related to both hyperactivity and impulsivity and containing items such as ‘I have trouble waiting in line or taking turns with others’. The remaining 12 items belong to the *ADHD index*, capturing features of ADHD that are not included in the DSM diagnostic criteria, such as ‘sometimes my attention narrows so much that I am oblivious to everything else; other times it’s so broad that everything distracts me’. A *DSM-IV ADHD total symptom* score can be calculated by summing the scores of the inattention and hyperactivity/impulsivity subscales. The CAARS-S:SV has good internal consistency and inter-rater reliability (31), high criterion validity and moderate concurrent validity (32).

T-scores were calculated for each subscale using the scores of the standardization sample provided in the technical manual consisting of non-clinical adults in the same age range and of the same sex. Subscale T-scores equal or above 65 indicate clinically elevated symptoms according to the technical manual (30). Therefore, participants without an ADHD diagnosis who had T-scores below 65 on all CAARS-S:SV subscales were excluded from all analyses. Although the CAARS-S:SV was included in the surveys at all three time points, only the baseline scores were used in the current study. For details about the ADHD symptom scores at all time points, see Haijen and colleagues (7).

#### 2.3.5. Trait mindfulness

To assess trait mindfulness, the 15-item Five Facet Mindfulness Questionnaire (FFMQ-15) was used (33). The FFMQ-15 consists of five subscales, with every three items capturing one aspect of mindfulness. The first subscale *observation* refers to attending to or noticing internal and external experiences and contains items such as ‘when I take a shower or a bath, I stay alert to the sensations of water on my body’. *Description* assesses the ability to put one’s experiences into words and contains items such as ‘I’m good at finding words to describe my feelings’. *Acting with awareness* captures the ability to attend to the present moment activity, without behaving automatically and while the attention is focused elsewhere. This subscale contains three reverse-phrased items such as ‘I do not pay attention to what I’m doing because I’m daydreaming, worrying, or otherwise distracted’. *Non-judging of inner experience* describes accepting and not evaluating emotions and thoughts as good or bad and contains three reverse-phrased items such as ‘I believe some of my thoughts are abnormal or bad and I should not think that way’. *Non-reactivity to inner experiences* involves detachment of emotions and thoughts allowing them to come and go without being carried away by them and contains items such as ‘when I have distressing thoughts or images, I “step back” and am aware of the thought or image without getting taken over by it’ (34). Items were rated on a five-point Likert scale ranging from 1 (never or very rarely true) to 5 (very often or always true). Subscale scores range from 3 to 15 and can be summed to achieve a total score ranging from 15 to 75. Reverse-phrased items were recoded. High scores on each mindfulness facet reflect a higher level of mindfulness. The FFMQ-15 showed adequate internal consistency and did not differ from the long form of the FFMQ in terms of convergent validity (34).

#### 2.3.6. Personality

To assess personality traits, the 10-item version of the Big Five Inventory (BFI; (35)) was included. This short questionnaire contains two items, of which one is reverse-phrased, describing each of the five Big Five personality traits. Example items include for *extraversion* ‘I see myself as someone who is outgoing, sociable’, for *agreeableness* ‘I see myself as someone who is generally trusting’, for *conscientiousness* ‘I see myself as someone who does a thorough job’, for *neuroticism* ‘I see myself as someone who gets nervous easily’, and for *openness* ‘I see myself as someone who has an active imagination’. Items are rated on a five-point Likert scale ranging from 1 (disagree strongly) to 5 (agree strongly). Reverse-phrased items were recoded. The scores of the two items belonging to one subscale were summed and divided by two to achieve an average subscale score ranging from 1 to 5. The BFI-10 has shown to be an adequate assessment of personality, with good validity and reliability metrics (35).

#### 2.3.7. MD substance and dose

Through short daily surveys starting from the day after the baseline survey until the 4-week time point, participants were asked each day if they had taken a microdose that day (yes/no), if yes, what substance (LSD, novel lysergamides (e.g., 1P-LSD), psilocybin/psilocin (magic mushrooms/truffles), mescaline (e.g., san pedro), or free text) and dose they took (free text).

### 2.4. Statistical analyses

All data were entered into the statistical program IBM SPSS Statistics version 26. Descriptive statistics were used to describe the demographic variables, information regarding psychiatric and physical diagnoses, previous experience with psychedelics, experience with meditation/mindfulness, and drug types and doses that were used for MD during the study. Linear mixed model (LMM) analysis was used to assess changes in personality traits and mindfulness after 2 and 4 weeks of MD compared to baseline. All LMMs contained the within-subject factor time [three levels: baseline (0 W), 2- (2 W), and 4-week (4 W) time point]. The binary factors, medication use and comorbidity were included as covariates in all LMMs. The fixed part of the models consisted of time, medication use, and comorbidity, and the interaction terms Time x Medication use and Time x Comorbidity.

To test whether MD increased mindfulness, the total score of the FFMQ-15 and the subscale scores (i.e., observation, description, acting with awareness, non-judging of inner experience, and non-reactivity to inner experiences) were included as dependent variables into separate LMMs. To control for a potential effect of recent experience with mindfulness and/or meditation, the LMMs were run again with the addition of the variable recent mindfulness as a covariate, by including recent mindfulness and the interaction between time and recent mindfulness as additional fixed factors in the LMMs.

To test whether MD affected personality traits, the subscale scores of the BFI-10 (i.e., conscientiousness, neuroticism, extraversion, agreeableness, and openness) were included as dependent variables in separate LMMs.

To find the best-fitting covariance structure for each LMM, Akaike’s information criterion (AIC) was used. Restricted maximum-likelihood (REML) estimation was used to estimate missing data. In case of significant main effects, pairwise comparisons between time points were conducted and corrected for multiple comparisons using Bonferroni correction. A significance level of 0.05 was used. Effect sizes were described by partial eta squared (*η*_p_^2^) values, where 0.01, 0.09, and 0.25 were considered small, medium, and large, respectively (36). Effect sizes were calculated using an online effect size calculator.^2^

## 3. Results

### 3.1. Demographic information and history of substance use

In total, 247 participants completed the baseline survey. Fast responses (i.e., below 50% of the median response time) were visually checked for inconsistencies in responding, leading to the exclusion of two respondents. Furthermore, 12 respondents who did not have an ADHD diagnosis had T-scores lower than 65 on all CAARS-S:SV subscales at baseline and were therefore excluded from all analyses. Sample sizes included in the analyses were 233, 66, and 44 at the three time points, respectively. Half of the sample at baseline consisted of female participants (*n* = 117; 50.2%), over 80 percent resided in Europe (*n* = 193; 82.8%), and most participants completed a tertiary level of education (*n* = 170; 73%). The most common daily occupations included computer/office work (*n* = 57; 24.5%), studying (*n* = 43; 18.5%), and working with people (*n* = 39; 16.7%). The majority of the sample at baseline had used a psychedelic substance at least once before (*n* = 191; 82%), see Table 1 for previously used psychedelic substances in both full and low doses at baseline. For demographic information at the 2- and 4-week time points, see Haijen and colleagues (7).

**Table 1:** Previous experience with psychedelic substances in full/regular psychedelic doses and low/micro doses of the whole sample (*n* = 233).

	Full or MD	Full dose experience	Microdose experience
*n* (%)	191 (82.0%)	178 (76.4%)	101 (43.3%)
Psilocybin/psilocin (e.g., magic mushrooms, truffles)	165 (70.8%)	152 (65.2%)	77 (33.0%)
LSD	111 (47.6%)	102 (43.8%)	43 (18.5%)
Ayahuasca	40 (17.2%)	38 (16.3%)	4 (1.7%)
DMT	38 (16.3%)	37 (15.9%)	5 (2.1%)
Salvia divinorum	24 (10.3%)	24 (10.3%)	1 (0.4%)
Novel lysergamides (e.g., 1P-LSD, ALD-52)	18 (7.7%)	16 (6.9%)	10 (4.3%)
Mescaline	14 (6.0%)	13 (5.6%)	3 (1.3%)
5-MeO-DMT	8 (3.4%)	7 (3.0%)	1 (0.4%)
Ibogaine	2 (0.9%)	2 (0.9%)	1 (0.4%)

### 3.2. Psychiatric and physical diagnoses

The majority of the sample at baseline had a current diagnosis of a psychiatric, neurological, or physical disorder (*n* = 166; 71.2%). ADHD was the most common diagnosis (*n* = 159; 68.2%), followed by depression (*n* = 44; 18.9%), anxiety disorder (*n* = 39; 16.7%), and PTSD (*n* = 17; 7.3%). Of the respondents diagnosed with ADHD (*n* = 159), had 86 individuals at least one comorbid diagnosis (54.1%). Depression (*n* = 42; 48.8%), anxiety disorder (*n* = 36; 41.9%), PTSD (*n* = 16; 18.6%), and dyslexia (*n* = 12; 14%) were the most reported comorbid diagnoses alongside ADHD. Of the respondents without an ADHD diagnosis, seven (9.5%) reported having a current diagnosis of a psychiatric, neurological, and/or physical disorder other than ADHD. Almost half of the participants who were diagnosed with ADHD, received this diagnosis when they were aged between 20 and 29 years old (*n =* 71; 44.7%), almost one quarter were between 30 and 39 years old (*n =* 38; 23.9%), 15 percent were between 10 and 19 years old (*n =* 24; 15.1%), and 10 percent were older than 40 when receiving the ADHD diagnosis (*n =* 17; 10.1%). Almost 14 percent of those diagnosed with ADHD never used any prescribed ADHD medication (*n =* 22; 13.8%) and one-third were using prescribed ADHD medication during the study (n = 53; 33.3%), with amphetamines (*n =* 27; 50.1%) and methylphenidate (n = 21; 39.6%) being the most common types of medication. The majority of ADHD-diagnosed individuals had tried prescribed ADHD medication in the past but stopped using it prior to baseline (*n =* 84; 52.8%). The most often reported reasons for discontinuing the prescribed ADHD medication were: because of physical side effects (*n =* 53; 63.1%), because of psychological side effects (*n =* 51; 32.1%), and because it did not relieve the symptoms (*n =* 17; 10.7%).

### 3.3. Experience with mindfulness/meditation

Over 80% (*n =* 194, 83.3%) of the sample indicated having experience with the practice of meditation/mindfulness. Of these, most respondents indicated to have used a mobile application for meditation/mindfulness purposes (*n =* 97; 50%), followed by watching Youtube videos (*n =* 84; 43.3%), following sessions at a retreat (*n =* 69; 35.6%), and/or following online courses (*n =* 51; 26.3%). Of those with meditation/mindfulness experience, the majority practised meditation/mindfulness for the last time within the past 7 days (*n =* 108; 55.7%), 30 respondents between 1 and 4 weeks ago (15.5%), 34 respondents more than 1 month ago (17.5%) and the remaining 22 respondents practised meditation/mindfulness more than 1 year ago for the last time (11.3%).

### 3.4. MD substance and dose

See Table 2 for the substances and average doses used during the study by the respondents who reported information through daily reports (*n =* 117; 50.2%). Two participants switched from using LSD or a novel lysergamide (e.g., 1P-LSD, ALD-52) to psilocybin-containing mushrooms/truffles, and one participant switched from psilocybin-containing mushrooms/truffles to LSD during the study.

**Table 2:** Substances and doses used during the study.

	Frequency (% of 117)	Mean dose (SD)
Psilocybin-containing mushrooms, truffles^1^	91 (77.8)	722 mg (485.5)
Novel lysergamides (e.g., 1P-LSD, ALD-52)	14 (12.0)	17.5 μg (31.1)
LSD	11 (9.5)	12 μg (6.4)
Ayahuasca	1 (0.9)	–

1 No further data was collected on whether psilocybin-containing mushrooms/truffles were dried or fresh.

### 3.5. Trait mindfulness

#### 3.5.1. FFMQ-15 total score

**Figure 1:**
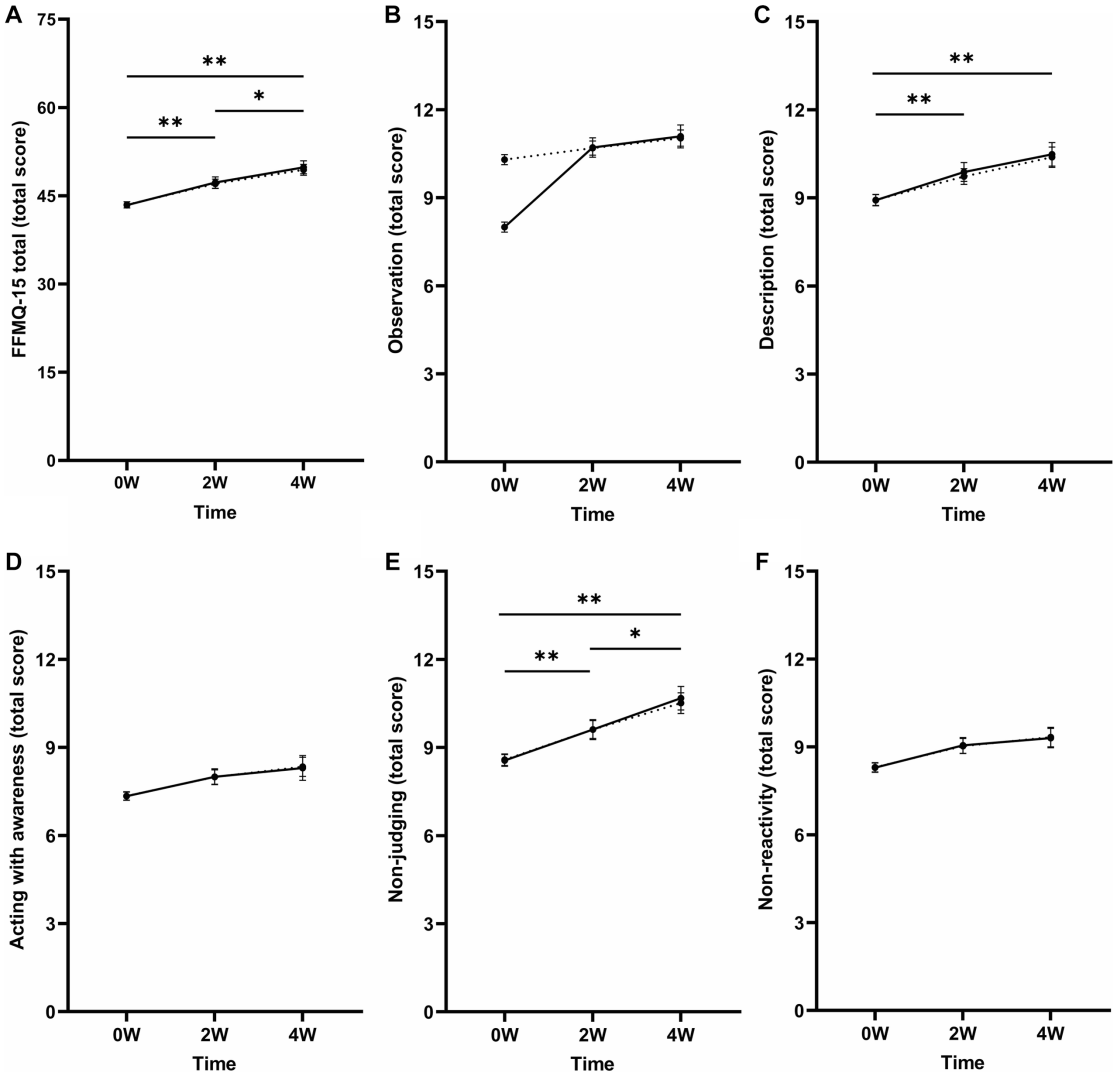
Mean total scores of the FFMQ-15 **(A)** total and the separate subscales **(B)** observation, **(C)** description, **(D)** acting with awareness, **(E)** non-judging of inner experiences, **(F)** and non-reactivity of inner experiences at baseline (0 W) and 2 (2 W) and 4 weeks (4 W) after MD. Corrected means (dotted line) are presented alongside the raw means (solid line) to aid the interpretation of the change in FFMQ-15 scores after including the covariates in the model (i.e., medication use, comorbidity, and recent mindfulness). The dotted and solid lines overlap when the means did not change after including the covariates. Mean differences that remained significant after including the recent mindfulness variable and after correction for multiple testing are indicated with asterisks (*). Error bars represent mean ± SEM. *p < 0.05; **p < 0.001.

Compound symmetry was used as a covariance structure for this LMM. A main effect of time was found on the total score of the FFMQ-15 [*F*_(2, 120.9)_ = 19.29, *p* < 0.001, *η*_p_^2^ = 0.24]. Pairwise comparisons showed that total mindfulness scores were higher at both 2 W (Δ2W–0 W = 3.62, *p* < 0.001) and 4 W (Δ4W–0 W = 5.97, *p* < 0.001) compared to baseline. Scores were also higher at 4 W compared to 2 W (Δ4W–2 W = 2.35, *p* = 0.043) (see Figure 1A). Further, a significant interaction between time and medication use was found [*F*_(2, 120.8)_ = 3.77, *p* = 0.026, *η*_p_^2^ = 0.06]. The estimates of fixed effects showed that scores were lower at 2 W in respondents using conventional ADHD medication compared to respondents who did not use conventional medication (*β* = −5.16, *p* = 0.009). The difference in scores was not significant at baseline (*β* = −0.25, *p* = 0.847) or 4 W (*β* = −1.41, *p* = 0.544). No interaction between comorbidity and time was found [*F*_(2, 121.3)_ = 0.35, *p* = 0.704, *η*_p_^2^ = 0.01].

When including the variable recent mindfulness in the model, the effect of time [*F*_(2, 120.9)_ = 11.18, *p* < 0.001, *η*_p_^2^ = 0.16], including the pairwise comparisons, and the interaction between time and medication use [*F*_(2, 121.2)_ = 3.30, *p* = 0.040, *η*_p_^2^ = 0.05] remained significant. There was no interaction between time and recent mindfulness [*F*_(2, 121.1)_ = 0.18, *p* = 0.833, *η*_p_^2^ = 0.00].

#### 3.5.2. Observation

Compound symmetry was used as the covariance structure for this model. The LMM showed an effect of time on the FFMQ-15 observation scores [*F*_(2, 119.1)_ = 3.10, *p* = 0.049, *η*_p_^2^ = 0.05]. Pairwise comparisons showed that scores were higher at 4 W compared to baseline (Δ4W–0 W = 0.75, *p* = 0.008). Scores did not differ between 2 W and baseline (Δ2W–0 W = 0.40, *p* = 0.158) and 2 W and 4 W (Δ4W–2 W = 0.35, *p* = 0.545). Furthermore, no interaction effect between time and medication use [*F*_(2, 119.5)_ = 1.80, *p* = 0.170, *η*_p_^2^ = 0.03] or time and comorbidity [*F*_(2, 119.8)_ = 0.01, *p* = 0.989, *η*_p_^2^ = 0.00] was found.

When including the variable recent mindfulness in the model, the effect of time was no longer significant [*F*_(2, 117.1)_ = 0.33, *p* = 0.718, *η*_p_^2^ = 0.00; see Figure 1B]. No interaction between time and recent mindfulness was found [*F*_(2, 117.2)_ = 1.96, *p* = 0.146, *η*_p_^2^ = 0.02].

#### 3.5.3. Description

The First-order autoregressive (AR1) covariance structure was the best fit for this model. A main effect of time was found on the FFMQ-15 scores [*F*_(2, 117.1)_ = 7.75, *p* < 0.001, *η*_p_^2^ = 0.12; see Figure 1C]. Pairwise comparisons revealed that scores were higher at 2 W (Δ2W–0 W = 0.81, *p* = 0.002) and 4 W (Δ4W–0 W = 1.42, *p* < 0.001) compared to baseline. The scores did not differ between the 2 W and 4 W (Δ4W–2 W = 0.61, *p* = 0.121). No interaction between time and medication use [*F*_(2, 119.4)_ = 1.86, *p* = 0.161, *η*_p_^2^ = 0.03] or time and comorbidity [*F*_(2, 119.6)_ = 0.69, *p* = 0.505, *η*_p_^2^ = 0.00] was found.

When including the variable recent mindfulness in the model, the effect of time remained significant [*F*_(2, 117.1)_ = 6.87, *p* = 0.002, *η*_p_^2^ = 0.11] and the results of the pairwise comparisons remained unchanged. Additionally, no interaction effect between time and recent mindfulness was found [*F*_(2, 117.2)_ = 2.32, *p* = 0.103, *η*_p_^2^ = 0.02].

#### 3.5.4. Acting with awareness

An unstructured covariance structure was used for this model. A main effect of time was found on the FFMQ-15 acting with awareness scores [*F*_(2, 51.3)_ = 4.70, *p* = 0.013, *η*_p_^2^ = 0.16]. Pairwise comparisons showed that scores were higher at both 2 W (Δ2W–0 W = 0.64, *p* = 0.029) and 4 W (Δ4W–0 W = 1.01, *p* = 0.005) compared to baseline. Scores did not differ between 2 W and 4 W (Δ4W–2 W = 0.37, *p* = 0.490). Furthermore, the interaction effects between time and medication use [*F*_(2, 51.7)_ = 1.09, *p* = 0.343, *η*_p_^2^ = 0.04] and time and comorbidity [*F*_(2, 51.4)_ = 0.23, *p* = 0.792, *η*_p_^2^ = 0.01] were not significant.

When including the variable recent mindfulness in the model, the time effect was no longer significant [*F*_(2, 50.7)_ = 2.88, *p* = 0.066, *η*_p_^2^ = 0.10; see Figure 1D]. Further, the interaction effect between time and recent mindfulness was not significant [*F*_(2, 50.3)_ = 0.49, *p* = 0.616, *η*_p_^2^ = 0.10].

#### 3.5.5. Non-judging of inner experience

Compound symmetry was used as a covariance structure. A main effect of time was found on the FFMQ-15 non-judging of inner experience scores [*F*_(2, 115.3)_ = 13.40, *p* < 0.001, *η*_p_^2^ = 0.19; see Figure 1E]. Pairwise comparisons showed that scores were higher at 2 W (Δ2W–0 W = 1.04, *p* < 0.001) and 4 W (Δ4W–0 W = 1.94, *p* < 0.001) compared to baseline. Scores were also higher at 4 W compared to 2 W (Δ4W–2 W = 0.90, *p* = 0.036). Further, a significant interaction between time and medication use was found [*F*_(2, 115.9)_ = 4.82, *p* = 0.010, *η*_p_^2^ = 0.08]. Estimates of fixed effects showed that non-judging of inner experience scores were lower at 2 W (*β* = −2.32, *p* = 0.002), not at baseline (*β* = −0.23, *p* = 0.643) or 4 W (*β* = −0.90, *p* = 0.306) for respondents using conventional medication alongside MD compared to respondents not using conventional medication. No interaction between time and comorbidity was found [*F*_(2, 116.4)_ = 0.79, *p* = 0.456, *η*_p_^2^ = 0.01].

When including the variable recent mindfulness in the model, the main effect of time [*F*_(2, 115.0)_ = 8.46, *p* < 0.001, *η*_p_^2^ = 0.13], including the pairwise comparisons, and the interaction between time and medication use [*F*_(2, 115.2)_ = 4.55, *p* = 0.013, *η*_p_^2^ = 0.07] remained significant. Additionally, no interaction effect between time and recent mindfulness was found [*F*_(2, 115.2)_ = 0.00, *p* = 0.999, *η*_p_^2^ = 0.00].

#### 3.5.6. Non-reactivity to inner experience

First-order autoregressive (AR1) was used as a covariance structure for this LMM. A main effect of time was found on the FFMQ-15 non-reactivity to inner experience scores [*F*_(2, 131.0)_ = 5.24, *p* = 0.006, *η*_p_^2^ = 0.07]. Pairwise comparisons showed higher scores at both 2 W (Δ2W–0 W = 0.73, *p* = 0.011) and 4 W (Δ4W–0 W = 1.05, *p* = 0.007) compared to baseline. Scores did not differ between 2 W and 4 W (Δ4W–2 W = 0.32, *p* = 0.960). No interaction between time and medication use [*F*_(2, 132.6)_ = 1.86, *p* = 0.159, *η*_p_^2^ = 0.03] or time and comorbidity [*F*_(2, 133.4)_ = 0.06, *p* = 0.945, *η*_p_^2^ = 0.00] was found.

After including the recent mindfulness variable in the model, the main effect of time was no longer significant [*F*_(2, 132.0)_ = 2.43, *p* = 0.092, *η*_p_^2^ = 0.04; see Figure 1F]. No interaction between time and recent mindfulness was found [*F*_(2, 132.2)_ = 0.22, *p* = 0.806, *η*_p_^2^ = 0.00].

### 3.6. Personality traits

#### 3.6.1. Conscientiousness

**Figure 2:**
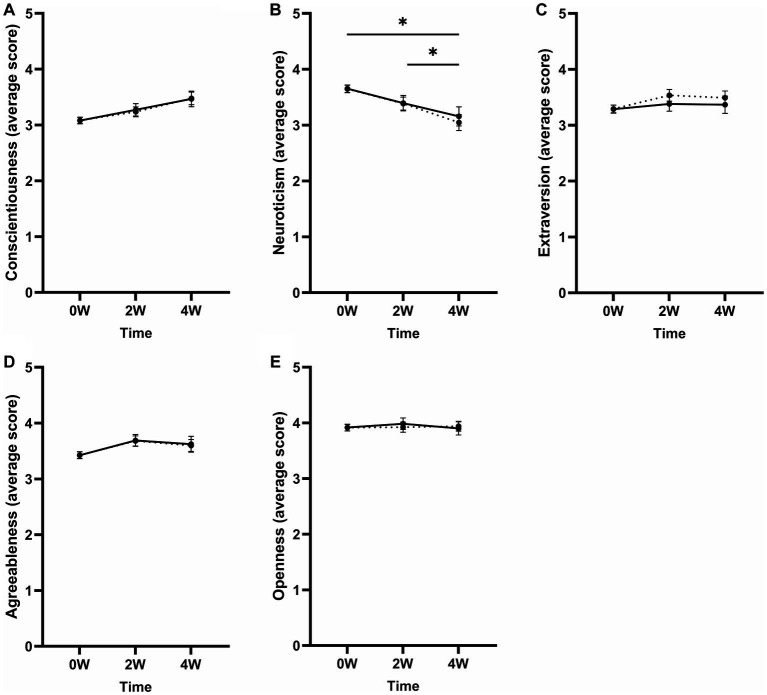
Mean scores of the BFI-10 subscales **(A)** conscientiousness, **(B)** neuroticism, **(C)** extraversion, **(D)** agreeableness, and **(E)** openness to experience at baseline (0 W), 2 (2 W), and 4 weeks (4 W) after MD. Corrected means (dotted line) are presented alongside the raw means (solid line) to aid the interpretation of the change in BFI-10 scores after including the covariates in the model (i.e., medication use and comorbidity). The dotted and solid lines overlap when the means did not change after including the covariates. Error bars represent mean ± SEM. Effects that remained significant after correction for multiple testing are indicated by an asterisk (*). *p < 0.05; **p < 0.001.

A First-order autoregressive (AR1) covariance structure was used for this model. A main effect of time on BFI-10 conscientiousness scores was found [*F*_(2, 136.3)_ = 3.77, *p* = 0.025, *η*_p_^2^ = 0.05]. Bonferroni-corrected pairwise comparisons showed that scores were higher at 4 W compared to baseline (Δ4W–0 W = 0.40, *p* = 0.002). Scores did not differ between baseline and 2 W (Δ2W–0 W = 0.16, *p* = 0.159) or between 2 W and 4 W (Δ4W–2 W = 0.24, *p* = 0.060; see Figure 2A). No interactions between time and medication use [*F*_(2, 137.9)_ = 0.69, *p* = 0.502, *η*_p_^2^ = 0.01] and time and comorbidity [*F*_(2, 138.3)_ = 0.10, *p* = 0.909, *η*_p_^2^ = 0.00] were found. After correcting for multiple testing, the effect found on conscientiousness scores was no longer significant.

#### 3.6.2. Neuroticism

An Ante-Dependence: First-Order covariance structure was the best fit for the model. A significant effect of time on BFI-10 neuroticism scores was found [*F*_(2, 60.8)_ = 7.19, *p* = 0.002, *η*_p_^2^ = 0.19]. Scores were lower at 4 W compared to baseline (Δ4W–0 W = −0.60, *p* < 0.001) and 2 W (Δ4W–2 W = −0.34, *p* = 0.014; see Figure 2B). Scores were not significantly lower at 2 W compared to baseline (Δ2W–0 W = −0.26, *p* = 0.056). No interactions between time and medication use [*F*_(2, 60.2)_ = 1.08, *p* = 0.350, *η*_p_^2^ = 0.034] and time and comorbidity [*F*_(2, 60.4)_ = 0.23, *p* = 0.796, *η*_p_^2^ = 0.01] were found. The variable comorbidity did have a main effect on the neuroticism scores [*F*_(1, 103.6)_ = 6.65, *p* = 0.011, *η*_p_^2^ = 0.06], showing higher neuroticism scores for respondents with comorbid diagnoses alongside the ADHD diagnosis compared to respondents without comorbidities alongside ADHD at baseline (*β* = 0.38, *p* = 0.007) and 2 W (*β* = 0.51, *p* = 0.048), but not at 4 W (*β* = 0.58, *p* = 0.064).

#### 3.6.3. Extraversion

A compound symmetry covariance structure was the best fit for this model. A main effect of time was found on the BFI-10 extraversion scores [*F*_(2, 123.8)_ = 3.58, *p* = 0.031, *η*_p_^2^ = 0.06]. Corrected pairwise comparisons showed that scores were higher at 2 W compared to baseline (Δ2W–0 W = 0.24, *p* = 0.039) (see Figure 2C). Scores did not differ between baseline and 4 W (Δ4W–0 W = 0.20, *p* = 0.249), or between 2 W and 4 W (Δ4W–2 W = −0.04, *p* > 0.999). No interaction between time and medication use [*F*_(2, 124.6)_ = 1.55, *p* = 0.217, *η*_p_^2^ = 0.02] or time and comorbidity [*F*_(2, 125.0)_ = 0.43, *p* = 0.652, *η*_p_^2^ = 0.01] was found. Comorbidity had an overall effect on extraversion scores [*F*_(1, 316.8)_ = 8.49, *p* = 0.004, *η*_p_^2^ = 0.03]. Respondents with at least one comorbid diagnosis alongside ADHD had lower scores at baseline (*β* = −0.48, *p* = 0.001) and 4 W (*β* = −0.62, *p* = 0.019), but not at 2 W (*β* = −0.38, *p* = 0.103). After correcting for multiple testing, the effect found on extraversion was no longer significant.

#### 3.6.4. Agreeableness

Compound symmetry was chosen as the covariance structure for this LMM. No effect of time was found on BFI-10 agreeableness scores [*F*_(2, 129.5)_ = 2.05, *p* = 0.133, *η*_p_^2^ = 0.03; see Figure 2D]. Further, no interaction between time and medication use [*F*_(2, 130.3)_ = 2.16, *p* = 0.120, *η*_p_^2^ = 0.03] or time and comorbidity was found [*F*_(2, 130.8)_ = 0.28, *p* = 0.755, *η*_p_^2^ = 0.00].

#### 3.6.5. Openness

An unstructured covariance matrix was the best fit for this model. The time effect on BFI-10 openness scores was not significant [*F*_(2, 51.4)_ = 0.42, *p* = 0.662, *η*_p_^2^ = 0.02; see Figure 2E]. No interaction between time and medication use [*F*_(2, 51.8)_ = 0.27, *p* = 0.766, *η*_p_^2^ = 0.01] or time and comorbidity [*F*_(2, 51.7)_ = 1.07, *p* = 0.350, *η*_p_^2^ = 0.04] was found.

## 4. Discussion

The current study aimed to investigate the effects of MD on mindfulness and personality traits in individuals diagnosed with ADHD and individuals without an ADHD diagnosis, who experienced severe ADHD complaints. In line with the expectations, mindfulness was increased after 2 weeks of MD compared to baseline and was further increased 2 weeks later. All facets of mindfulness (i.e., observation, description, acting with awareness, non-judging of inner experience, and non-reactivity to inner experience) were increased 4 weeks after MD compared to baseline. However, when taking recent mindfulness into account, only description and non-judging of inner experience remained significantly increased at both 2 and 4 weeks. Furthermore, the personality trait neuroticism was decreased after 4 weeks of MD compared to baseline. Extraversion and conscientiousness were increased after 2 and 4 weeks of MD compared to baseline, respectively, but these effects did not survive correction for multiple testing. The remaining personality traits agreeableness and openness remained unchanged after MD initiation. Using conventional medication alongside MD or having comorbid diagnoses next to the ADHD diagnosis did not influence the change in any of the mindfulness or personality traits after 4 weeks of MD compared to baseline.

At baseline, the current sample showed on average a lower total mindfulness score as well as lower scores for each mindfulness facet compared to the mean scores of general population samples (37, 38). This lower score was expected, based on the population, if it were not that the majority of the current sample did have previous experience in meditation and/or mindfulness within 7 days prior to completing the baseline measure. After 4 weeks of MD, the current sample reported total and subscale mindfulness scores that were similar to the mean scores of general population samples, except for the acting with awareness subscale (37, 38). The finding that mindfulness was enhanced in MD individuals is in line with previous studies reporting this association (6, 11), but in contrast to one previous prospective MD study that did not find any changes in mindfulness after 6 weeks of MD (13). Polito and Stevenson (13) used the Mindful Attention Awareness Scale (MAAS) to assess mindfulness making a one-on-one comparison not possible. The discrepancy between the findings by Polito and Stevenson (13) and the current study could potentially be explained by different sample characteristics as they excluded individuals with certain mental disorders, such as mood and anxiety disorders. It may be that MD effects are more pronounced in clinical populations as there is more room to detect changes. However, it might be concluded that the MD-induced changes in mindfulness are most pronounced for non-judging of inner experience and description as these were the only two subscales that still showed MD-induced changes when controlling for recent mindfulness/meditation experience.

At baseline, the personality traits conscientiousness and extraversion were on average lower compared to the mean BFI-10 scores of general population samples (39, 40). In contrast, neuroticism, agreeableness, and openness were at baseline on average higher compared to the mean BFI-10 scores of general population samples (39, 40). The reported baseline conscientiousness, extraversion and neuroticism scores were in line with previously reported associations between ADHD and personality traits (20, 21, 25). In contrast, agreeableness and openness were relatively high in the current sample at baseline while previous studies reported a negative or no relationship between ADHD symptoms and agreeableness and openness, respectively (20, 23, 24, 26). After 4 weeks of MD, conscientiousness and extraversion scores were on average higher than the mean scores of the general population sample reported by Rettenberger and colleagues (39) but remained below the mean scores reported by Blüml amd colleagues (40). Although neuroticism significantly decreased within the current sample after 4 weeks of MD, scores remained on average higher than the mean scores reported by general population samples (39, 40). The decrease in neuroticism reported here is consistent with two previous prospective MD studies (12, 29). In contrast, Polito and Stevenson (13) found an increase in neuroticism after MD. However, as was discussed by Dressler and colleagues (29), the increase in neuroticism might be seen in individuals who have little to no experience with MD or psychedelics in general. The majority of the current sample (80%) had previous experiences with psychedelics and were perhaps well-prepared regarding what to expect, preventing an increase in neuroticism. Based on previous studies, it was expected that the current sample would increase on the remaining four personality traits after MD. However, the increase in conscientiousness seen after 4 weeks and the increase in extraversion seen after 2 weeks of MD in the current study did not survive correction for multiple testing and additionally, the effect sizes were small. Agreeableness and openness to experience did not change at all after MD compared to baseline in the current study, contrasting results from earlier MD studies (12, 28). The lack of findings here might be because of a ceiling effect since the current sample scored already high on these personality traits at baseline.

A limitation of the current study design was the lack of experimental control. Uncertain and perhaps inaccurate reports of the doses and substances used limited the possibility to make inferences about what exactly participants had taken and whether differences in substance and/or dose could have led to different effects on mindfulness and/or personality traits in adults experiencing ADHD symptoms. On the other hand, a strength of the employed design was the ecological validity as it captured MD-induced changes that occur in individuals who are MD on their own initiative, a practice we know is prevalent in current Western societies. Additionally, a strength of the current design compared to retrospective and cross-sectional designs was the use of multiple time points, enabling a comparison of mindfulness and personality after MD initiation to the participant’s baseline traits. Thereby, it allows making causal inferences with less uncertainty compared to retrospective and cross-sectional studies. In contrast, a disadvantage of including multiple time points was the large drop-out rate, which could lead to biased results, since participants who perhaps did not have a pleasant MD experience stopped prematurely, potentially creating a more “positive” picture of the effect of MD in ADHD than is truly the case. Related to this, the large drop-out led to a relatively small sample size at the 4-week time point (*n =* 44). The finding that the changes in the mindfulness facets observation, acting with awareness, and non-reactivity to inner experience were no longer significant after including an additional, third, covariate could also be a consequence of a reduction in power or overfitting of the model as in general, more observations per predictor lead to more reliable estimates (41).

Future research should investigate whether the MD-induced effects on mindfulness and neuroticism are long-lasting by including follow-up measurements after several months post-MD. Furthermore, the effects of MD on adult ADHD should be tested in a controlled setting, to ensure drug and dose uniformity, including a (placebo) control group. However, it is important to consider that lab-based measures generally have low ecological validity, and it is therefore also a pressing need to start developing measures that are ecologically valid and sensitive to the effects induced by low doses of psychedelics. Additionally, it would be of interest to compare the effects of MD on mindfulness to a non-pharmacological mindfulness intervention as well as the combination of MD with a mindfulness intervention in adults experiencing ADHD symptoms, to test whether effects induced by MD are comparable to a mindfulness intervention, or whether the combination of both elicits synergistic effects. Lastly, given the previously mixed findings regarding the effects of MD on the personality trait neuroticism, it would be of interest to test whether an increase in neuroticism induced by MD is related to the lack of previous experience with psychedelics. If that is the case, this is an important consideration for future clinical applications and emphasizes the importance of preparation prior to treatment with MD for ADHD, but also other patient populations that might benefit from MD.

To conclude, the current study found positive changes in mindfulness, specifically the mindfulness facets description and non-judging of inner experience, and in the personality trait neuroticism after 4 weeks of MD compared to baseline in adults with an ADHD diagnosis or severe ADHD symptoms. These positive changes might be reflective of the therapeutic properties of MD in this patient population. Future (placebo) controlled studies are warranted to confirm these findings.

## Data availability statement

The original contributions presented in the study are included in the article/supplementary material, further inquiries can be directed to the corresponding author.

## Ethics statement

The studies involving humans were approved by The Ethics Review Committee of Psychology and Neuroscience at Maastricht University. The studies were conducted in accordance with the local legislation and institutional requirements. The participants provided their written informed consent to participate in this study.

## Author contributions

KK and EH designed the study. EH collected and analyzed the data under supervision of KK and PH. EH wrote the first version of the article. All authors contributed to the article and approved the submitted version.

## Footnotes

^1^ www.microdosinginstitute.com

^2^ www.effect-size-calculator.herokuapp.com
